# Evaluation of Radial Peripapillary Capillary Density in G6PD Deficiency: An OCT Angiography Pilot Study

**DOI:** 10.3390/jcm11123282

**Published:** 2022-06-08

**Authors:** Rita Serra, Giuseppe D’Amico Ricci, Stefano Dore, Florence Coscas, Antonio Pinna

**Affiliations:** 1 Department of Biomedical Sciences, University of Sassari, Viale San Pietro 43, 07100 Sassari, Italy; 2 Istituto di Ricerca Genetica e Biomedica (IRGB), CNR, Cittadella Universitaria di Cagliari, 09042 Monserrato, Italy; 3 Centre Ophtalmologique de l’Odeon, 113 bd Saint Germain, 75006 Paris, France; coscas.f@gmail.com; 4 Ophthalmology Unit, Department of Medical, Surgical, and Experimental Sciences, University of Sassari, 07100 Sassari, Italy; giuseppedamicoricci@icloud.com (G.D.R.); stefanodore@hotmail.com (S.D.); apinna@uniss.it (A.P.)

**Keywords:** glucose-6-phosphate-dehydrogenase deficiency, optic nerve head, optical coherence tomography angiography, radial peripapillary capillary, vascular density

## Abstract

Glucose-6-phosphate-dehydrogenase (G6PD) deficiency is an inherited enzymatic disorder causing hemolytic anemia. The purpose of this pilot study was to compare vascular density (VD) values of the radial peripapillary capillary (RPC) plexus in G6PD-deficient and G6PD-normal men, using optical coherence tomography angiography (OCTA). Methods: 46 G6PD-deficient men and 23 age-matched male controls were included. A complete ophthalmological evaluation, consisting of slit-lamp biomicroscopy, best-corrected visual acuity, intra-ocular pressure measurement, structural optical coherence tomography, and OCTA scanning of the optic nerve head, was performed. The en-face angioflow images were carefully analyzed and the VD values of the RPC plexus were measured using the AngioAnalytics™ software embedded in the OCTA device. Medical conditions, including systemic hypertension, hypercholesterolemia, and diabetes mellitus, were also investigated. Results: G6PD-deficient eyes showed higher values of VD in all peripapillary sectors, but a statistical significance (*p* = 0.03) was reached only in the infero-temporal sector. There were no significant differences in terms of hypercholesterolemia, systemic arterial hypertension, and diabetes mellitus between the two study groups. Conclusion: Results show that VD values of the RPC plexus are higher in G6PD-deficient men than in G6PD-normal subjects, but a statistically significant difference was found only in the inferior temporal sector. Overall, our preliminary findings support the hypothesis that the RPC layer of G6PD-deficient men consists of a denser vascular network, which may contribute to offering protection against ocular atherosclerotic vasculopathies.

## 1. Introduction

With a worldwide prevalence of approximately 500 million people affected, glucose-6-phosphate-dehydrogenase (G6PD) deficiency is an inherited enzymatic disorder causing hemolytic anemia [1].

G6PD is a ubiquitous cytoplasmatic enzyme implicated in the regulation of carbon flow through the pentose phosphate pathway, essential for cell protection against oxidative stress. G6PD plays a crucial role in the conversion of glucose-6-phosphate into 6-phosphogluconate, a biochemical reaction associated with the simultaneous synthesis of reduced nicotinamide adenine dinucleotide phosphate (NADPH), essential for the proper functioning of glutathione reductase, an enzyme that transforms glutathione disulfide (GSSG) into reduced glutathione (GSH) [2,3]. Hemizygous males have uniformly deficient erythrocytes, whereas heterozygous females have mosaic populations of G6PD-deficient and normal red blood cells because of random inactivation of the X chromosome.

In red blood cells, NADPH production and, therefore, protection against oxidative stress are strongly related to G6PD activity [2]. Consequently, in G6PD-deficient patients, the ingestion of agents with oxidant properties, such as broad bean (*Vicia faba*) or some drugs (e.g., quinine derivatives, sulphonamides, and several anti-inflammatory agents), may trigger a decreased availability of GSH that may cause oxidation of hemoglobin and peroxidation of membrane lipids, thus leading to erythrocyte lysis and jaundice, which are typical clinical manifestations of hemolytic anemia [4].

In Sardinia (Italy), a major Mediterranean island, G6PD deficiency represents a real public health issue, since over 10% of the population shows the G6PD-deficient genotype [4].

It has been supposed that the high prevalence of this inherited enzymopathy may derive from the natural selection induced by Plasmodium falciparum malaria, endemic in Sardinia until the 1950s. In fact, G6PD deficiency, associated with increased oxidative stress in red blood cells, impairs plasmodium growth, offering protection against the development of severe malaria [5].

Previous studies have highlighted that G6PD deficiency seems also to be associated with a decreased risk of developing several systemic and ophthalmological conditions, including cardiovascular and cerebrovascular diseases, colorectal cancer, retinal vein occlusion (RVO), and nonarteritic anterior ischemic optic neuropathy (NA-AION) [2,4,6,7]. G6PD deficiency also shows a trend for protection against severe proliferative diabetic retinopathy (PDR) [8]. Although some hypotheses have been proposed, how exactly G6PD deficiency may be involved in protecting against the occurrence of RVO, NA-AION, and perhaps severe PDR is still not known [4].

The retina is a highly metabolic tissue requiring tight regulation of blood perfusion and redox homeostasis for normal functioning. Cumulative oxidative stress causes damage to the retinal microvasculature and ganglion cells. Evidence from animal models indicates that endothelial Nox2, an NADPH-dependent enzyme, contributes to age-related capillary rarefaction in nervous tissue by increasing the production of reactive oxygen species [9]. Theoretically, G6PD deficiency, characterized by decreased NADPH production and, consequently, Nox2 activity, might be associated with a better capillary network.

In the past, our knowledge and understanding of the pathophysiological mechanisms underlying retinal vascular diseases were limited to traditional angiography, an invasive imaging technique allowing only a two-dimensional en-face analysis of the retinal vasculature [10]. The recent introduction of optical coherence tomography angiography (OCTA) in clinical practice has revolutionized our approach to retinal and optic nerve head (ONH) assessment [11]. Indeed, this novel imaging technique generates high-resolution images of retinal and ONH plexuses [11,12]. Specifically, OCTA has made possible the detailed in vivo visualization of the radial peripapillary capillary (RPC) plexus, which is not feasible with traditional imaging techniques [11,13].

Recent research has focused on the RPC plexus, which plays a crucial role in the nourishment of retinal ganglion cells and, as a result, is involved in the pathogenesis of several retinal disorders [14,15,16]. The embedded OCTA software, by automatically providing quantitative parameters (e.g., vascular density [VD]), allows for the objective evaluation of the blood flow and circulation in the RPC plexus, opening new areas of interesting research [16].

The number of studies assessing the RPC plexus of the ONH is limited [13]. Furthermore, we are unaware of any previous published report investigating the RPC plexus in G6PD-deficient patients. Therefore, the aim of the present study was to determine the VD values of the RPC plexus in G6PD-deficient and G6PD-normal patients and ascertain whether, or not, there were significant differences in terms of peripapillary microvascular changes between the two study groups.

## 2. Materials and Methods

This was a case–control study, enrolling 46 consecutive G6PD-deficient men and 23 apparently healthy G6PD-normal male controls, who were examined at the Ophthalmology Unit, Department of Medical, Surgical, and Experimental Sciences, University of Sassari, Sassari, Italy, from January to June 2017. Women were excluded due to the small number of homozygote subjects with a total lack of erythrocyte G6PD activity.

This observational study was performed according to the tenets of the Declaration of Helsinki for research involving human subjects. Institutional ethics review board approval was obtained. Each participant was given detailed information and provided written consent before inclusion.

The inclusion criteria for cases were male gender, G6PD deficiency, clear ocular media, and intra-ocular pressure (IOP) <20 mm Hg. Exclusion criteria included a history of ocular trauma, retinal and optic disc disease, uveitis, high myopia (≥6 diopters), intraocular surgery (apart from phacoemulsification performed at least 6 months before the study entry), diabetes mellitus, thalassemia trait, and kidney failure.

Apart from the presence of normal G6PD activity, the inclusion and exclusion criteria for the controls were the same as those for the cases.

Blood samples were collected from each participant. Erythrocyte G6PD activity was analyzed using a quantitative assay (G6PD/6PGD, Biomedic snc, Sassari, Italy), as described previously [4].

All participants underwent a full ophthalmological examination and optic disc imaging. This included measurement of best-corrected visual acuity (BCVA), slit-lamp biomicroscopy with dilated indirect ophthalmoscopy, Goldmann applanation tonometry, B-scan OCT (Topcon OCT-2000), and OCTA (AngioVue XRTVue Avanti, Optovue, Fremont, CA, USA). 

The OCTA scanning area was centered on the ONH. The AngioVue disc mode automatically segmented the ONH into four layers: ONH, vitreous, RPC plexus, and choroid. We assessed 4.5 × 4.5 mm OCTA scans of the RPC plexus, which extended from the inner limiting membrane (ILM) to 100 µm under the ILM, which we set as the lower boundary.

Each scan was reviewed to confirm the correct segmentation and sufficient image quality, as confirmed by the signal strength index (>55), and was repeated when necessary.

Furthermore, VD values of the RPC plexus, provided using the AngioAnalytics software embedded in the OCTA device, were recorded and compared with those of healthy eyes.

Color-coded perfusion maps were automatically generated using the flow density map software AngioAnalytics, which provided the VD values of the nine different peripapillary sectors (grid-based flow density) of the entire ONH scan. In brief, bright red stood for perfused vessel density of >50%, dark blue meant no perfusion, and intermediate perfusion densities were color-coded accordingly.

Medical conditions such as diabetes mellitus, systemic hypertension, and hypercholesterolemia were assessed. Specifically, participants were classified as hypertensive if they were receiving antihypertension drugs or if their blood pressure was >140 mm Hg systolically or >90 mm Hg diastolically (according to the WHO/International Society of Hypertension). Participants were considered to be diabetic if they were under treatment for insulin- or non-insulin-dependent diabetes mellitus, or if their fasting plasma glucose level was >126 mg/dL and/or their plasma glucose level was >200 mg/dL 2 h after a 75 g oral glucose load in a glucose tolerance test (as defined by the WHO). Hypercholesterolemia was defined by the intake of lipid-lowering drugs or a fasting plasma cholesterol level of >220 mg/dL [4].

The statistical software package Minitab 19 for macOS (Statistical Software. [Computer software]. State College, PA, USA: Minitab, Inc. www.minitab.com; accessed on 5 June 2022) and/or Stata 14 for Mac OS X (StataCorp. 2015. Stata Statistical Software: Release 14. College Station, Texas, U.S.: StataCorp LP) were used to perform data processing, summaries, and analyses. Missing data were not replaced. Summary statistics, including the number of subjects (N), number of observations (Obs), mean, median, standard deviation (SD), minimum (Min), and maximum (Max), were calculated for continuous variables. Frequencies and percentages were calculated for categorical data. A Wilcoxon rank-sum test was performed between groups for each variable. A multivariate analysis of covariance (MANCOVA) test was used to examine statistical differences between groups with regards to the flow density maps of the RPC plexus. Statistical tests were two-sided at the 0.05 significance level with 95% confidence intervals. 

## 3. Results

Our study included 46 eyes of 46 G6PD-deficient men (mean age 43.93 ± 15.79 years) and 23 eyes of 23 apparently healthy G6PD-normal men (mean age 45.30 ± 18.02 years). The mean BCVA was 0.07 ± 0.12 LogMAR in the G6PD-defient group and −0.1 ± 0.1 LogMAR in the controls, which is not a statistically significant difference. In the G6PD-deficient group, three eyes were pseudophakic, whereas there were five pseudophakic eyes in the control group. The refractive error (spherical equivalent) was −0.69 ± 2.01 diopters in G6PD-deficient patients and −0.55 ± 1.05 diopters in the controls, which again is not a significant difference. No significant differences were found as well in terms of hypercholesterolemia, systemic hypertension, and diabetes between the two groups. All demographic and clinical data of both study groups are shown in Table 1.

The VD values of the RPC plexus in G6PD-deficient and G6PD-normal individuals are summarized in Table 2. G6PD-deficient eyes showed higher values of VD in all peripapillary sectors, but a statistical significance (*p* = 0.03) was reached only in the infero-temporal sector.

**Figure 1:**
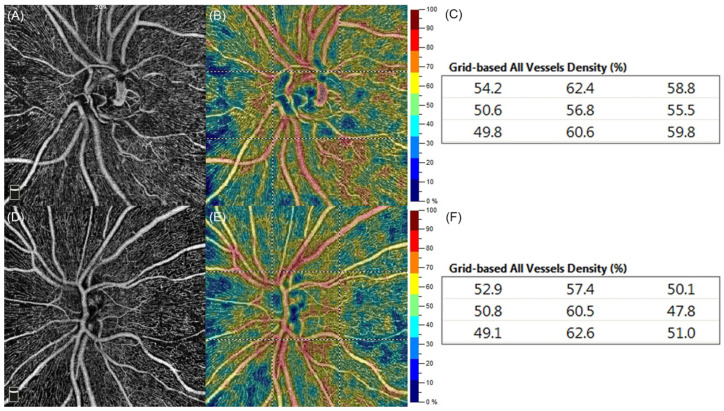
Optical coherence tomography angiography (OCTA) of the radial peripapillary capillary (RPC) plexus in Glucose-6-Phosphate-Dehydrogenase (G6PD)-deficient and G6PD-normal individuals of the same age (55 years). RPC en-face angiograms (**A**,**D**), corresponding color-coded perfusion maps (**B**,**E**), and grid-based vessels density (VD; **C**,**F**) from a G6PD-deficient man (**A**–**C**) and a G6PD-normal control (**D**–**F**). In (**C**), VD values are higher than in (**F**) in six out of nine quadrants.

Representative images of the RPC plexus in G6PD-deficient and G6PD-normal individuals are shown in Figure 1.

## 4. Discussion

In this case–control study, we compared ONH findings in G6PD-deficient and G6PD-normal individuals. Specifically, we focused on the VD of the RPC plexus as automatically detected by OCTA. Interestingly, in G6PD-deficient subjects, the VD values of the RPC plexus were higher, reaching a statistically significant difference in the inferior temporal sector (*p* = 0.03).

RPCs are the innermost layer of capillaries surrounding the ONH and extending straight along the course of the retinal nerve fiber layer (RNFL) to the posterior pole [13,16,17,18,19]. Light microscope observations have highlighted that RPCs do not derive from ONH arterioles or retinal arteries [18]; rather, they seem to arise from deeper arterial vessels located in the ganglion cell layer or in the outer RNFL and then extend to the superficial RNFL, where they form parallel rows, with rare anastomoses and bifurcations [13,14].

Recent OCTA reports on the appearance of RPCs have confirmed that these capillaries have a peculiar distribution and arrangement that runs along the paths of the major temporal vessels up to 4–5 mm from the ONH, and that they form the most superficial vascular layer located within the RNFL [16,17,18,19]. RPCs, which are easy to identify via OCTA by their typical features, seem to be specialized in the nourishment of the RNFL, especially the most superficial nerve fibers, which subserve the peri-central visual field [15].

Bjerrum scotoma is a peri-central visual field defect strongly related to selective damage to RPCs, which are more vulnerable to increased IOP than other retinal capillaries, probably because of their peculiar distribution and vascular pattern [14,16]. Therefore, it has been supposed that RPCs may represent the first damage site in glaucomatous eyes [13,16].

OCTA has revolutionized the vascular imaging approach towards the ONH, allowing for visualization and qualitative/quantitative assessment of the RPC plexus. Noteworthy recent research has focused on the study of the RPC plexus because injuries at this level seem to play a key role, not only in glaucoma, but also in retinal vascular disorders (e.g., RVO, PDR) [15,18].

Histopathological evidence indicates that occlusion of linear blood vessels parallel to RNFL axons, corresponding to the RPC plexus, translates into the occurrence of flame-shaped hemorrhages and cotton wool spots due to stagnant axonal flow in the RNFL [20]. The exact pathogenetic mechanism behind RVO remains elusive; however, intraluminal thrombus formation has been associated with venous stasis, endothelial damage, and hypercoagulability. A strong correlation between RVO and typical atherosclerosis risk factors, including systemic hypertension, hypercholesterolemia, and diabetes mellitus, has widely been documented [4,21,22].

In two former studies on Sardinian patients with RVO and NA-AION, the frequency of G6PD deficiency was found to be significantly lower than expected, suggesting that G6PD-deficient subjects may have a decreased risk of developing RVO and NA-AION [4,7]. Why and how G6PD deficiency may offer protection against RVO and NA-AION is still a matter of debate. Batetta et al. [23] have demonstrated that G6PD-deficient patients show decreased cholesterol synthesis and esterification due to peculiar alterations in lipid metabolism, which is likely to be responsible for reduced cholesterol accumulation in the arteries of these subjects. Therefore, the slower progression of the atherosclerotic process in G6PD-deficient patients may translate into a reduced risk of atherosclerotic vascular disorders, including RVO and NA-AION. Furthermore, this theory may explain why G6PD-deficient patients show low mortality rates from cardiovascular and cerebrovascular diseases [2].

In our study, G6PD-deficient men had significantly higher VD values of the RPC plexus than age-matched G6PD-normal controls, suggesting the presence of a denser vascular network in the former. Theoretically, this OCTA finding may represent an advantage in protection against retinal vascular disorders.

The NADPH oxidase (Nox) family catalyzes the reduction of O_2_ to reactive oxygen species, coupled with NADPH oxidation. Seven isoforms of Nox (Nox1-5 and Duox1-2) have been identified so far. Among them, Nox2 is highly expressed in cells throughout the central nervous system and retina, including endothelial cells [8,24]. There is evidence from mouse studies that endothelial Nox2 activity increases with age in the brain and contributes to age-related capillary rarefaction [8]. Since Nox2 is an NADPH-dependent enzyme, G6PD-deficient individuals might experience reduced endothelial Nox2 activity and reduced age-related capillary rarefaction.

The balance between the activity of nitric oxide synthase (NOS), an NAPDH-dependent enzyme, and concentrations of glutathione (GSH), a physiological NO scavenger, may play a crucial role in preventing the development of vascular disorders [25,26]. It is not known whether NOS and GSH are out of balance in G6PD-deficient subjects; however, experimental evidence indicates that G6PD deficiency may lead to decreased NO production [27]. In G6PD deficiency, it is possible that the combined action of NO and GSH may be responsible for inhibiting low-density lipoprotein esterification, smooth muscle cell proliferation, and platelet aggregation, which may lead to vessel dilation and a slow-down of the atherosclerotic process.

The present study has some important limitations, principally related to the small number of eyes examined. In total, 69 male patients were enrolled, and the G6PD-deficient group was chosen to be twice as large as the control group. Furthermore, no women were included in this study due to a lack of homozygote female subjects available. Nevertheless, we are unaware of any previously published report assessing the VD of the RPC plexus in G6PD-deficient and G6PD-normal individuals.

In conclusion, our results show that G6PD-deficient men have higher VD values in the RPC plexus, although a statistically significant difference was found only in the inferior temporal sector (*p* = 0.03). A denser vascular network at this level, together with decreased cholesterol synthesis and esterification, may contribute to explaining why G6PD-deficient men seem to have a reduced risk of ocular vascular diseases, such as RVO and NA-AION. Further larger studies are necessary to confirm these preliminary findings and establish the potential protective role of G6PD deficiency against ocular diseases.

## Figures and Tables

**Table 1:** Demographic and clinical data of glucose-6-phosphate-dehydrogenase (G6PD)-deficient patients and G6PD-normal controls.

	G6PD-Deficient Subjects	G6PD-Normal Controls
Total eyes, n	46	23
Total patients, n	46	23
Age, mean ± SD (years)	43.93 ± 15.79	45.30 ± 18.02
BCVA, mean ± SD (LogMAR)	0.07 ± 0.12	−0.10 ± 0.10
Refractive error, mean ± SD (spherical equivalent)	−0.69 ± 2.01	−0.55 ± 1.05

Categorical variables are shown as numbers. Continuous variables are shown as mean ± standard deviation (SD). BCVA = best-corrected visual acuity.

**Table 2:** Radial peripapillary capillary (RPC) vascular density (VD) values in glucose-6-phosphate-dehydrogenase (G6PD)-deficient patients and G6PD-normal controls.

Peripapillary SECTORS	G6PD-Deficient Subjects	G6PD-Normal Controls	*p* Value
Supero-temporal	57.71 ± 4.27	56.49 ± 4.29	>0.05
Supero-middle	58.43 ± 4.08	58.08 ± 3.35	>0.05
Supero-nasal	50.02 ± 4.68	49.33 ± 5.37	>0.05
Intermediate-temporal	55.56 ± 3.99	55.30 ± 3.68	>0.05
Intermediate-nasal	50.13 ± 4.50	50.10 ± 4.00	>0.05
Inferior-temporal	58.75 ± 4.79	57.06 ± 4.27	**0.03**
Inferior-middle	59.79 ± 4.38	59.03 ± 4.14	>0.05
Inferior-nasal	46.75 ± 4.53	45.79 ± 4.17	>0.05

Continuous variables are shown as mean ± standard deviation (SD).

## Data Availability

Not applicable.

## References

[1] Luzzatto L., Poggi V., Orkin S.H., Nathan D.G., Ginsburg D., Look A.T., Fisher D.E., Lux S.E. (2009). Glucose-6-phosphate dehydrogenase deficiency. Nathan and Oski’s Hematology of Infancy and Childhood.

[2] Cocco P., Todde P., Fornera S., Manca M.B., Manca P., Sias A.R. (1998). Mortality in a cohort of men expressing the glucose-6-phosphate dehydrogenase deficiency. Blood.

[3] Beutler E. (1994). G6PD deficiency. Blood.

[4] Pinna A., Carru C., Solinas G., Zinellu A., Carta F. (2007). Glucose-6-Phosphate Dehydrogenase Deficiency in Retinal Vein Occlusion. Investig. Opthalmol. Vis. Sci..

[5] Siniscalco M., Bernini L., Latte B., Motulsky A.G. (1961). Favism and Thalassæmia and their Relationship to Malaria. Nature.

[6] Dore M.P., Davoli A., Longo N., Marras G., Pes G.M. (2016). Glucose-6-phosphate dehydrogenase deficiency and risk of colorectal cancer in Northern Sardinia: A retrospective observational study. Medicine.

[7] Pinna A., Solinas G., Masia C., Zinellu A., Carru C., Carta A. (2008). Glucose-6-Phosphate Dehydrogenase (G6PD) Deficiency in Nonarteritic Anterior Ischemic Optic Neuropathy in a Sardinian Population, Italy. Investig. Opthalmol. Vis. Sci..

[8] Pinna A., Contini E.L., Carru C., Solinas G. (2013). Glucose-6-Phosphate Dehydrogenase Deficiency and Diabetes Mellitus with Severe Retinal Complications in a Sardinian Population, Italy. Int. J. Med. Sci..

[9] Fan L.M., Geng L., Cahill-Smith S., Liu F., Douglas G., Mckenzie C.A., Smith C., Brooks G., Channon K.M., Li J.M. (2022). Nox2 contributes to age-related oxidative damage to neurons and the cerebral vasculature. J Clin. Investig..

[10] Serra R., Coscas F., Boulet J.F., Cabral D.R., Lupidi M., Coscas G.J., Souied E.H. (2020). Predictive Activation Biomarkers of Treatment-Naive Asymptomatic Choroidal Neovascularization in Age-Related Macular Degeneration. Retina.

[11] Spaide R.F., Klancnik J.M., Cooney M.J. (2015). Retinal Vascular Layers Imaged by Fluorescein Angiography and Optical Coherence Tomography Angiography. JAMA Ophthalmol..

[12] Serra R., Sellam A., Coscas F., Bruyère E., Sieiro A., Coscas G.J., Souied E.H. (2018). Evaluation of pseudophakic cystoid macular edema using optical coherence tomography angiography. Eur. J. Ophthalmol..

[13] Scoles D., Gray D.C., Hunter J.J., Wolfe R., Gee B.P., Geng Y., Masella B.D., Libby R.T., Russell S., Williams D.R. (2009). In-Vivo imaging of retinal nerve fiber layer vasculature: Imaging histology comparison. BMC Ophthalmol..

[14] Alterman M., Henkind P. (1968). Radial peripapillary capillaries of the retina. II. Possible role in Bjerrum scotoma. Br. J. Ophthalmol..

[15] Kornzweig A.L., Eliasoph I., Feldstein M. (1968). Selective Atrophy of the Radial Peripapillary Capillaries in Chronic Glaucoma. Arch. Ophthalmol..

[16] Mansoori T., Sivaswamy J., Gamalapati J.S., Balakrishna N. (2017). Radial Peripapillary Capillary Density Measurement Using Optical Coherence Tomography Angiography in Early Glaucoma. J. Glaucoma.

[17] Michaelson I. (1954). Retinal Circulation in Man and Animals.

[18] Henkind P. (1967). Radial peripapillary capillaries of the retina. I. Anatomy: Human and comparative. Br. J. Ophthalmol..

[19] Yu P.K., Balaratnasingam C., Xu J., Morgan W.H., Mammo Z., Han S., MacKenzie P., Merkur A., Kirker A., Albiani D. (2015). Label-Free Density Measurements of Radial Peripapillary Capillaries in the Human Retina. PLoS ONE.

[20] Ip M., Hendrick A. (2018). Retinal Vein Occlusion Review. Asia Pac. J. Ophthalmol. (Phila).

[21] Clarkson J.G., Ryan S.J. (2001). Central retinal vein occlusion. Medical Retina.

[22] Fekrat S., Finkelstein D., Ryan S.J. (2001). Branch retinal vein occlusion. Medical Retina.

[23] Batetta B., Bonatesta R.R., Sanna F., Putzolu M., Mulas M.F., Collu M., Dessì S. (2002). Cell growth and cholesterol metabolism in human glucose- 6-phosphate dehydrogenase deficient lymphomononuclear cells. Cell Prolif..

[24] Kowluru R.A., Radhakrishnan R., Mohammad G. (2021). Regulation of Rac1 transcription by histone and DNA methylation in diabetic retinopathy. Sci. Rep..

[25] Wallace M. (1996). NADPH diaphorase activity in activated astrocytes representing inducible nitric oxide synthase. Methods Enzym..

[26] Li H., Poulos T.L. (2005). Structure–function studies on nitric oxide synthases. J. Inorg. Biochem..

[27] Stanton R.C. (2012). Glucose-6-phosphate dehydrogenase, NADPH, and cell survival. IUBMB Life.

